# Influence of tailing dust contamination in drinking water on the health status of goats in subtropical Regions

**DOI:** 10.1007/s11250-024-04163-z

**Published:** 2024-10-02

**Authors:** William Ntshete Serakalala, Zwelethu Mfanafuthi Mdletshe

**Affiliations:** Department of Agricultural Economics and Animal Production, Faculty of Science and Agriculture, University of Limpopo, Private Bag X1106, Sovenga, 0727 South Africa

**Keywords:** Tailing dust, River systems, Drinking water, Seasonality, Goat health

## Abstract

The contamination of river systems by tailing dust remains a constraint to goat productivity in communal farming systems. A cross-sectional study was conducted to investigate how resource-limited households in subtropical regions assessed the effects of tailing dust contamination in drinking water on the health status of goats. In a study conducted in the Ba-Phalaborwa Local Municipality, 200 households from two villages were randomly selected and interviewed using a questionnaire. Forty-eight percent (*n* = 96) of the households were located in contaminated areas, while 52% (*n* = 104) were from uncontaminated areas. The study found that poor water quality, caused by tailing dust contamination, as well as a high incidence of diseases, were key factors affecting goat productivity. Water contamination was most severe during the hot and cool dry seasons. It was also noted that goats rely on freshwater as their primary source of supplemental water during dry seasons. Urine colour, oedema of the eyelids, and kid survival were indicators for assessing the health status of goats. Nominal binary logistic regression revealed that water contamination was 2.96 more likely to be reported by youth compared to elderly members. Farmers who received informal education were 37 times more likely to report contamination than those who received formal education. High kid mortality as a health status indicator was 50 times less likely to be reported in uncontaminated areas. Intervention strategies for reducing the adverse health effects of tailing dust in contaminated areas should focus primarily on the health of goats during dry seasons.

## Introduction

More than 60% of all goats are kept by rural households in arid and semi-arid agroecological zones, accounting for approximately 96% of the world's total goat population (Andre Mataveia et al. 2021). Goats are important to resource-limited households as they provide meat, generate additional income when sold as live animals, and produce manure (Mdletshe et al. 2018). Goats are also used for performing religious and cultural rituals and assessing the status of community members among other social roles. Even though goats offer these benefits, one of the primary challenges to goat production is poor water quality (Mdletshe et al. 2017; Runa et al. 2019; Ndlela et al. 2022).

Poor water quality is an important limiting factor of goat production under communal production systems in subtropical regions. In subtropical areas, goats face a major health problem due to drinking water contamination by tailing dust containing toxic heavy metals such as Arsenic (As), copper (Cu), lead (Pb), and zinc (Zn). This contamination is caused by the drying of water cover over mine tailings due to reduced mineral precipitation and increased evapotranspiration (Cleaver et al. 2021; Opara et al. 2022). Consuming water contaminated with heavy metals can lead to cell damage and various disorders, such as respiratory, gastrointestinal, central nervous system, renal, and liver disorders (Fashola et al. 2016; Wrzecińska et al. 2021). Symptoms may include changes in urine colour, eye membrane infections, and excessive salivation. Little to no information is available about the effects of tailing-contaminated water on goat health, despite the potential risks of heavy metal-contaminated water on birds and pets.

Seasonality and distance from the tailing dam both affect the levels of heavy metal contamination in surface water bodies (Kołacz et al. 2020). As a result of being exposed to toxic heavy metals in drinking water that is in contaminated areas, goats have to travel long distances in search of less contaminated water (Mseleku et al. 2020). Consumption of heavy metals has been associated with neurological, circulatory system, and reproductive diseases (Wrzecińska et al. 2021; Kowalczyk et al. 2022). The influence of tailing in the contamination of drinking water used by goats on productivity has not been documented and has an important consequence on the flock, nutritional management and general health status. Studies on the effects of drinking water contaminated with mine tailing dust on goats' productivity and health status have been limited, if any, because of the emphasis on cattle and other intensively managed livestock. This is due to the perception that goats can tolerate poor water quality (Mdletshe et al. 2017; Ndlela et al. 2022).

Understanding the beliefs of goat-owning households about tailing dust pollution and its impact on goat health will help policymakers develop sustainable solutions to enhance goat productivity and promote safe mining practices. Therefore, the objective of the study is to determine how households perceive the effect of tailing dust contamination on the health status of goats. It was assumed that households in contaminated and uncontaminated areas would experience the effect of tailing dust contamination on goat health status similarly.

## Materials and method

### Description of the study site

The study was conducted in two communities of Ba-Phalaborwa Local Municipality (23° 55′ 0′′ S, 30° 50′ 0′′ E), Limpopo Province, South Africa. Ba-Phalaborwa has a Subtropical steppe and is located at an altitude of 437 m above sea level. The climate receives summers that are long, hot, muggy, and partly cloudy and winters that are short, cool, dry, and clear. Average annual rainfall ranges between 96 and 6 mm per annum, the rainy season is between November and February. The warmest months are December and January with an average temperature of 34 $^\circ{\rm C}$ and the lowest average temperatures of 26 $^\circ{\rm C}$ in July. Goats browsed and grazed in communally land during the day and were housed in the afternoon.

### Water sampling

Twelve water samples in total (six from each zone) were collected using single-use polyethylene narrow-mouth bottles with screw caps. Each bottle was sterilized in the water that would be collected and kept in a cooler box with ice for testing of heavy metals at the University of Limpopo. Using inductively coupled plasma atomic emission spectrometry (ICPE-900) (ICP-MS, IRIS Advantages, USA), the amounts of chromium (Cr), copper (Cu), magnesium (Mg), and lead (Pb) were measured. The information was then used to assess and choose areas for questionnaire sampling that had access to both contaminated and uncontaminated water that goats use for drinking.

### Household selection and data collection

Households were selected based on their distance from the Bosveld Phosphate Mine (6 and 15 km). In Ga-Makhushane village, most water sources for goat drinking were contaminated by tailing dust, while in Ga-Mashishimale village, most water sources were not contaminated (Table 1). The target class of weaners and non-lactating does were made available by prioritising households with flocks of 20 or more goats.

**Table 1:** Physicochemical characteristics of water samples for goat drinking in areas contaminated and uncontaminated by tailing dust (minimum, maximum, mean, and standard error)

Parameters	Contaminated areas			Uncontaminated areas			Normal
	Minimum	Maximum	Mean ± STD	Minimum	Maximum	Mean ± STD	Maximum
pH	8.21	8.22	8.22 ± 0.01	7.97	8.07	8.01 ± 0.05	6.5–8
Dissolved Oxygen (mg/l)	18.3	18.4	18.3 ± 0.06	56.4	57.2	56.87 ± 0.42	6.5–8
Conductivity (μ/cm)	1	1.039	1.03 ± 0.02	0.583	0.583	0.58 ± 0.00	0–1.5
TDS (mg/l)	0.5773	0.773	0.70 ± 0.11	0.442	0.440	0.00	0–1000
Salinity (mg/l)	0.33	0.33	0.33 ± 0.00	0.33	0.33	0.00	0–4000
Chromium (mg/L)	25.7	27.3	26.6 ± 0.82	14.8	16.6	15.93 ± 0.99	0–1
Copper (mg/L)	0.509	0.541	0.53 ± 0.02	0.342	0.355	0.35 ± 0.01	0–0.5
Magnesium (mg/L)	97	99.3	98.23 ± 1.16	52.6	53.2	52.93 ± 0.31	0–500
Lead (mg/L)	4.33	4.68	4.56 ± 0.20	2.46	2.75	2.62 ± 0.15	0–0.01

A total of 200 households owning goats were interviewed with 96 from Ga-Makhushane and 104 from Ga-Mashishimale villages. Interviews were conducted in the presence of extension officers, an animal health technician, and elderly members of each community. Elderly members of the community, which were key informants validated the accuracy of data which was obtained from households that kept goats. Questionnaires were used to evaluate farmer perceptions of the health status of goats drinking non-contaminated and contaminated water with tailing dust. Enumerators were trained and interviews were conducted in Xitsonga Vernacular to get proper information. Interviews with every participant were conducted at their place of residence. The questionnaire covered household demographics, goat production and productivity, access to water and quality, and goat health.

### Statistical analysis

All data was analysed using the Statistical Analysis Software (SAS 2023). The chi-square test was employed to determine the relationships between household demographics and water quality. The general linear model procedure was used to determine the effect of water contamination on the composition and flock size. The chi-square test was also used to determine the relationship between household perceptions among farmers in contaminated and uncontaminated areas. The PDIFF option was used to create pairwise comparisons of the least square means. A nominal binary logistic regression (PROC LOGISTIC) was used to predict the odds of households’ perceived water contamination for goats between contaminated and uncontaminated areas. The variables that fitted in the logit model included age and literacy level.

The model used is shown below: $Ln \left[{P}_{i}\div {1-P}_{i}\right]= {\beta }_{0}+ {\beta }_{1}{X}_{1}+ {\beta }_{2}{X}_{2}+ {\beta }_{3}{X}_{3}+.. {\beta }_{t}{X}_{t}+ \varepsilon$

P is the probability of a farmer’s perceived goat health status, [${P}_{i}$/1 – ${P}_{i}$] is the odds ratio of a households perceived goat health status, ${\beta }_{0}$ is the intercept, ${\beta }_{1}$… ${\beta }_{t}$ are the regression coefficient of predictors, ${X}_{1}$…${X}_{t}$ are the predictor variables, and ε is the random residual error.

When computing each predictor (${\beta }_{1}$… ${\beta }_{t}$), the odds ratio for goat health status is interpreted as the proportion of households experiencing challenges with goat health against households that experience no challenges with goat health. A similar model is used to assess farmers' opinions on goat production methods, water accessibility, goat health, and the likelihood that farmers consider supplementing drinking water to dilute heavy metal contamination.

## Results

### Social demographic information and constraints to goat productivity

Household demographics for participants are shown in Table 2. Determination of water contamination was associated with literacy level (*P* ≤ 0.05) and livestock species (*P* ≤ 0.05). Most of the farmers (*P* ≤ 0.05) in contaminated (49%) and uncontaminated areas (38%) areas had not received formal education. Cattle herd size was generally smaller in contaminated (*P* ≤ 0.05) compared to uncontaminated areas. Poor water quality as a result of tailing dust contamination was the major constraint to goat productivity, followed by diseases, and water shortage (*P* ≤ 0.05) for households in contaminated compared to uncontaminated areas (Table 3).

**Table 2:** Comparing the socioeconomic status of farmers and the herd/flock sizes of different livestock species (least square means ± SE) kept per household, in areas with drinking water contaminated and uncontaminated by tailing dust

Parameters	Contaminated	Uncontaminated	X^2^	Significance
Gender				
Male	51	50	0.89	NS
Female	49	50		
Age (%)				
< 18	4	2	0.41	NS
18–30	20	14		
30–50	37	35		
> 50	39	49		
Education (%)				
No education	49	38	< 0.001	*
Primary	18	15		
Secondary	32	26		
Tertiary	1	21		
Training (%)				
Yes	2	0	0.16	NS
No	98	100		
Livestock Species				
Cattle	(1) 1.11 ± 0.11^a^	(2) 1.75 ± 0.10^b^		*
Goat	(2) 1.48 ± 0.07	(1) 1.50 ± 0.01		NS
Sheep	(3) 2.39 ± 0.22	(3) 1.94 ± 0.15		NS
Chicken	(4) 2.45 ± 0.13	(4) 2.44 ± 0.17		NS
Pigs	(5) 3.13 ± 0.26	(5) 2.50 ± 0.51		NS

The lower the rank (mean rank score), the greater its importance. Values in parentheses indicate means for ranks

*P ≤ 0.05; NS: Not significant (*P* ≥ 0.05)

**Table 3:** Ranking constraints on goat productivity for goats consuming water contaminated and uncontaminated by tailing dust

Variables	Contaminated	Uncontaminated	Significance
Water quality	1 (2.14)	6 (6.13)	*
Diseases	2 (2.43)	2 (1.92)	*
Water shortage	3 (2.94)	5 (5.49)	*
Theft	4 (4.51)	1 (1.67)	*
Parasites	5 (4.54)	3 (2.93)	*
Predators	6 (5.50)	4 (4.47)	*

The lower the rank (mean rank score), the greater its importance. Values in parentheses indicate means for ranks

*P ≤ 0.05; NS: Not significant (*P* ≥ 0.05)

### Water sources for drinking, seasonal water quality, and supplementation

There was an association (*P* ≤ 0.05) between water sources and quality (Table 4). Rainwater was generally reported to be the primary water source for goats, followed by dams, and rivers in both contaminated and uncontaminated areas. Furthermore, there was a relationship (*P* ≤ 0.05) between seasons and water quality (Table 5). Water contamination levels were reported to be high in the hot-dry season, followed by cool-dry, and rainy seasons in contaminated areas when compared to their counterparts.

**Table 4:** Comparing the drinking water sources of goats that consume water contaminated by tailing dust with those that consume uncontaminated water

Variables	Contaminated	Uncontaminated	Significance
Water sources			
Rainwater	1 (1.16)	1 (1.00)	*
Dam/pond	2 (2.31)	2 (1.01)	*
River	3 (2.55)	3 (1.99)	*

The lower the rank (mean rank score), the greater its importance. Values in parentheses indicate means for ranks

*P ≤ 0.05; NS: Not significant (*P* ≥ 0.05)

**Table 5:** Ranking seasonal challenges of water quality for goats consuming water contaminated and uncontaminated by tailing dust

Variables	Contaminated	Uncontaminated	Significance
Seasons			
Hot-dry	1 (1.42)	2 (1.73)	*
Cool-dry	2 (1.91)	1 (1.27)	*
Post-rainy	3 (2.95)	3 (3.04)	NS
Rainy	4 (3.72)	4 (3.93)	*

The lower the rank (mean rank score), the greater its importance. Values in parentheses indicate means for ranks

Values in parenthesis indicate means for ranks

*P ≤ 0.05; NS: Not significant (*P* ≥ 0.05)

### Seasonal water supplementation for goat drinking

There was an association (*P* ≤ 0.05) between water supplementation and seasonality (Table 6). Freshwater was reported to be the primary source of water supplementation, followed by dams/ponds, and rivers in contaminated areas compared to their counterparts. The main water supplement for goats was freshwater, which was more significant than greywater and boreholes in both rainy and hot-dry seasons (*P* ≤ 0.05).

**Table 6:** Ranking the effect of seasonal water supplements on goats consuming water contaminated and uncontaminated by tailing dust

Water sources	Rainy season		Hot-dry season		Significance level
	Contaminated	Uncontaminated	Contaminated	Uncontaminated	
Freshwater	1 (1.27)	1 (1.18)	1 (1.25)	1 (1.02)	*
Greywater	2 (1.90)	3 (3.08)	2 (1.97)	3 (3.05)	****
Borehole	3 (2.83)	2 (1.68)	3 (2.84)	2 (1.73)	****

The lower the rank (mean rank score), the greater its importance. Values in parenthesis indicate means for ranks

NS not significant (*P* ≥ 0.05)

*P ≤ 0.05; **P ≤ 0.01; ***P ≤ 0.001, ****P ≤ 0.0001

### Seasonal water contamination and health status of goats

There was an association (*P* ≤ 0.05) between seasonal water contamination and the health of goats (Table 7). In contaminated areas, urine colour was the most recognised health indicator for goats drinking water polluted by tailing dust across both seasons (*P* ≤ 0.0001). In uncontaminated areas, urine colour was ranked the primary indicator (*P* ≤ 0.0001) during rainy seasons when compared to hot-dry seasons. In areas where water is uncontaminated, kids’ survival was higher compared to contaminated areas (*P* ≤ 0.0001) across both rainy and hot-dry seasons.

**Table 7:** Ranking health status indicators for goats that consume tailing dust-contaminated and uncontaminated water between seasons

Health indicators	Rainy season		Hot-dry season		Significance level
	Contaminated	Uncontaminated	Contaminated	Uncontaminated	
Urine colour	1 (1.24)	3 (1.96)	1 (1.34)	4 (2.13)	****
Oedema of the eyelids	2 (1.63)	2 (1.75)	2 (1.67)	2 (1.85)	NS
Kids survival	3 (1.94)	1 (1.23)	3 (1.94)	1 (1.19)	****
Excessive salivation	4 (2.15)	4 (2.01)	4 (2.17)	3 (1.96)	NS

The lower the rank (mean rank score), the greater its importance. Values in parentheses indicate means for ranks

NS not significant (*P* > 0.05)

*P > 0.05; **P < 0.01; ***P < 0.001, ****P < 0.0001

### Water contamination on the health status of goats

Water contamination was associated (*P* ≤ 0.0001) with indicators for the health status of goats (Table 8). Urine colour was the primary health indicator followed by oedema of the eyelids, and kid mortality in contaminated areas compared to their counterparts.

**Table 8:** Ranking the health status indicators of goats consuming water contaminated and uncontaminated by tailings dust

Variables	Contaminated	Uncontaminated	Significance
Health indicator			
Urine colour	1 (1.28)	4 (2.00)	****
Oedema of the eyelids	2 (1.33)	2 (1.42)	NS
Kids mortality	3 (1.94)	1 (1.23)	****
Excessive salivation	4 (2.07)	3 (1.96)	NS
Loss of condition	5 (4.96)	5 (5.66)	****

The lower the rank (mean rank score), the greater its importance. Values in parentheses indicate means for ranks

Values in parenthesis indicates means for ranks *p ≤ 0.05; **p ≤ 0.01; ***p ≤ 0.001; ****p ≤ 0.0001; NS: Not significant (*p* ≥ 0.05)

### Odds ratio estimates for household demographics, water contamination, and health indicators for goats

Table 9 shows the probabilities of reporting water contamination for goat consumption. Male farmers were 1.2 times more likely to indicate tailing dust to contaminate water sources used by goats for drinking (P ≤ 0.05) than female farmers. When compared to farmers who received formal education, those with informal education were 37 times more likely to indicate tailing dust contamination in drinking water sources used by goats for drinking. High kid mortality as a health indicator that goats are drinking polluted water by tailing dust was 50 times less likely to occur in uncontaminated areas.

**Table 9:** Odds ratio estimates, lower (LCI) and upper (UCI) confidence intervals for the household perception of water contamination in areas with and without contamination about goat consumption

Predictors	Odds ratio	LCI	UCI	Significance
Age (youth vs older)	2.958	1.074	8.146	*
Education (Informal vs formal)	37.319	4.597	302.927	*
Kid mortality (High vs Low)	0.020	0.003	0.119	*
Water source (River vs rainwater)	18.999	0.843	428	NS
Loss of coordination (dull vs normal)	5.482	0.723	41.543	NS

Higher odds ratio estimates indicate greater differences in occurrence between levels of predictors. If the upper confidence interval > 1- * significantly difference; If the upper confidence interval < 1- not significantly (NS) different

## Discussion

Understanding goat farmers' beliefs about tailings dust polluting river systems and its impact on goat health can help policymakers develop sustainable plans to increase goat productivity, minimize negative effects, and promote safe mining methods. It was hypothesized that households with access to contaminated and non-contaminated drinking water would have similar farmer attitudes about the influence of tailing dust contamination on goat health. Education level affected understanding and awareness of tailing dust contamination's effect on drinking water sources and goat health in contaminated and uncontaminated areas. This finding is in agreement with that of Khapayi and Celliers (2016), who observed that farmers' knowledge of agricultural dynamics is limited by a lack of formal education.

The findings that goats' flock size was higher than other livestock species kept by farmers in contaminated areas could be associated with their tolerance to poor water quality and efficient water use (Silanikove 2000). These results are consistent with the findings reported by Mdletshe et al. (2017), who observed no difference between goats providing high- and low-salinity water regarding feed conversion efficiency, rectal temperature, and respiration rate. Further research must be done to determine how heavy metal contamination from tailings in river systems affects goat health.

Findings that suggest poor water quality is the primary constraint in contaminated areas could be explained by tailings polluting the soil, groundwater, and surface water sources that goats use for drinking. The results complement earlier studies from Serbia and Romania Tech 2013; Isvoran et al. 2021), which have shown that mining operations may contaminate surface water bodies that animals use for drinking through tailing dust dispersed into river systems. This can be explained by the fact that the cool-dry and hot-dry seasons are favourable periods for the production and dispersion of tailing dust because there is lower mineral precipitation and higher evapotranspiration. During the cool-dry and hot-dry seasons, there is less rainfall, less mineral precipitation, increased evapotranspiration, and higher saline levels in the surface drinking water that goats utilize to drink. Goat productivity is negatively impacted during this time, especially those owned by resource-limited households, which causes economic loss. In the current study, water quality is lower in the cool-dry and hot-dry seasons in contaminated areas. These findings are comparable with the findings of Cleaver et al. (2021) who reported variation in water pH and contamination levels based on seasonality and location from tailings.

During the hot-dry season, poor water quality is a major concern, as the lack of rainfall leads to a high concentration of tailings in water sources. This forces communal farmers to supplement with either freshwater or greywater. The findings that goat farmers in contaminated areas supplement with freshwater year-round are consistent with a previous study conducted in Tanzania by Mgode et al. (2021). The study showed that farmers with limited resources share the freshwater they use for themselves with their livestock. Cleaver et al. (2021) found that tailing dust contamination of water sources remains constant during rainy and post-rainy seasons. Providing freshwater helps reduce pollution's impact on goat health. The findings of this study showed that contaminated areas affected the supplementation of drinking water for goats. This could be further explained by the movement of water-soluble dust particles from tailing that contain high concentrations of heavy metals during the freeze–thaw and sublimation processes that occur in cold, windy conditions (Zwissler 2016).

In contaminated areas, goats drinking polluted water with tailing dust showed high kid mortality and changes in urine colour. These health indicators were associated with elevated levels of copper (Cu), chromium (Cr), and lead (Pb) in the water, which exceeded the recommended standards for drinking water (Table 1). The colour of a goat's urine can indicate its health when drinking polluted water with tailing dust. This aligns with Cushny's (2002) report, which found that high levels of lead in drinking water for livestock can cause kidney damage and result in highly concentrated or bloody urine. Contaminated drinking water, such as tailing dust, can increase kid mortality. Oyeyemi and Akusu (2005) found that high levels of Pb in water can cause gastrointestinal infections and diarrhoea, leading to kid mortality. Tailing contamination in goats' drinking water leads to a loss of condition. Mdletshe et al. (2017) observed that goats drinking poor water quality had reduced feed intake, resulting in a loss of condition due to low nutrient intake. Therefore, more information concerning the effect of tailings contamination in drinking water on the growth performance, physiological response, and blood chemistry of goats needs to be provided for effective management and control of tailings dust.

## Conclusion

Education level influenced farmers’ perception of dust contamination and its impact on the livestock species, and the number per household in the area. Tailing dust contamination was high during the hot-dry, cool-dry, and rainy seasons. In contaminated areas, fresh and household wastewater was the main source of water supplementation during the rainy and hot-dry seasons. For goats in these areas, urine colour, kids' mortality rate, and overall body condition score were indicators of the impact of drinking water contaminated by tailing dust. Mitigation strategies should target contaminated areas during cold, and hot-dry seasons. Further research should focus on goats' physiological response, blood haematology and chemistry in subtropical regions.

## Data Availability

The study's data will be made available upon request from the corresponding author (ZMM).

## References

[CR1] G Andre Mataveia C Visser A Sitoe (2021) Smallholder Goat Production in Southern Africa: A Review IntechOpen. 10.5772/intechopen.97792

[CR2] Cleaver AE, Jamieson HE, Rickwood CJ, Huntsman P (2021) Tailings dust characterization and impacts on surface water chemistry at an abandoned Zn-Pb-Cu-Au-Ag deposit. Appl Geochemistry 128:104927. 10.1016/j.apgeochem.2021.104927

[CR3] Cushny AR (2002) On Saline Diuresis. Physiol J 28(6):431. 10.1113/jphysiol.1902.sp000928 10.1113/jphysiol.1902.sp000928 PMC1540594 16992635

[CR4] DWAF (Department of Water Affairs and Forestry of the Republic of South Africa) (1996) South African water quality guidelines. Agricultural use: Livestock watering 5

[CR5] Fashola MO, Ngole-Jeme VM, Babalola OO (2016) Heavy metal pollution from gold mines: environmental effects and bacterial strategies for resistance. Int J Environ Res Public Health 13(11):1047. 10.3390/ijerph13111047 10.3390/ijerph13111047 PMC5129257 27792205

[CR6] Isvoran A, Roman DL, Dascalu D, Vlad-Oros B, Ciorsac A, Pitulice L, Jonovic R, Stevanovic Z, Ostafe V (2021) Human health effects of heavy metal pollution in the cross-border area of Romania and Serbia: a review. Ecol Chem Eng 28(3):365–388. 10.2478/eces-2021-0025

[CR7] Khapayi M, Celliers PR (2016) Factors limiting and preventing emerging farmers to progress to commercial agricultural farming in the King William’s Town area of the Eastern Cape Province. South Africa. SAJAE 44(1):25–41 (https://www.ajol.info/index.php/sajae/article/view/138546)

[CR8] Kołacz R, Spiak Z, Czaban S, Dobrzański Z, Opaliński S, Kowalska N, Cwynar P, Kupczyński R (2020) Copper and zinc concentrations in plant and animal raw materials collected in the vicinity of the żelazny most waste treatment tailings pond. J Elem 25(4):1423–1434. 10.5601/jelem.2020.25.1.1976

[CR9] Kowalczyk A, Wrzecińska M, Czerniaawska-Piatkowska E, Araújo JP, Cwynar P (2022) Molecular consequences of the exposure to toxic substances for the endocrine system of females. Biomed Pharmacother 155:113730. 10.1016/j.biopha.2022.113730 36152416 10.1016/j.biopha.2022.113730

[CR10] Mdletshe ZM, Chimonyo M, Marufu MC, Nsahlai IV (2017) Effects of saline water consumption on physiological responses in Nguni goats. Small Rumin Res 153:209–211. 10.1016/j.smallrumres.2017.06.019

[CR11] Mdletshe ZM, Ndlela SZ, Nsahlai IV, Chimonyo M (2018) Farmer perceptions on factors influencing water scarcity for goats in resource-limited communal farming environments. Trop Anim Health Prod 50:1617–1623. 10.1007/s11250-018-1603-x 29744725 10.1007/s11250-018-1603-x

[CR12] Mgode GF, Mhamphi GG, Massawe AW, RS, Machang’u (2021) Leptospira Seropositivity in Humans, Livestock and Wild Animals in a Semi-Arid Area of Tanzania. Pathog Dis 10(6):696. 10.3390/pathogens10060696 10.3390/pathogens10060696 PMC8230269 34205097

[CR13] Mseleku C, Ndlela SZ, Mkwanazi MV, Chimonyo M (2020) Health status of non-descript goats travelling long distances to water source. Trop Anim Health Prod 52:1507–1511. 10.1007/s11250-019-02094-8 31691914 10.1007/s11250-019-02094-8

[CR14] Ndlela SZ, Mdletshe ZM, Zindove TJ, Chimonyo M (2022) Do water shortages increase gastrointestinal nematode loads in Nguni does? Trop Anim Health and Prod 54(4):208. 10.1007/s11250-022-03171-1 10.1007/s11250-022-03171-1 35678955

[CR15] Opara CB, Blannin R, Ebert D, Frenzel M, Pollmann K (2022) Bioleaching of metal (loid) s from sulfidic mine tailings and waste rock from the Neves Corvo mine, Portugal, by an acidophilic consortium. Miner Eng 188:107831. 10.1016/j.mineng.2022.107831

[CR16] Oyeyemi MO, Akusu MO (2005) Retrospective study on some diseases causing mortality in West African Dwarf (Fouta djallon) goats during the first year of life: an eight-year study. Niger J Anim Prod 32(1):120–125

[CR17] Runa RA, Brinkmann L, Riek A, Hummel J, Gerken M (2019) Reactions to saline drinking water in Boer goats in a free-choice system. Animal 13(1):98–105 29679996 10.1017/S1751731118000800

[CR18] SAS (2023) Statistical analysis system user’s guide 9.4. Statistical Analysis System Institute, Inc. Cary, North Carolina, USA

[CR19] Silanikove N (2000) The physiological basis of adaptation in goats to harsh environments. Small Rumin Res 35(3):181–193. 10.1016/S0921-4488(99)00096-6

[CR20] Tech ET (2013) Determination of some heavy metals in wastewater and sediment of artisanal gold local mining site of Abare Area in Nigeria. J Environ Treat 1(3):174–182 (https://www.cabdirect.org/cabdirect/abstract/20143125444)

[CR21] Wrzecińska M, Kowalczyk A, Cwynar P, Czerniaawska-Piatkowska E (2021) Disorders of the Reproductive Health of Cattle as a Response to Exposure to Toxic Metals. Biol 10:882. 10.3390/biology10090882 10.3390/biology10090882 PMC8467698 34571759

[CR22] Zwissler BE (2016) Dust susceptibility at mine tailings impoundments: thermal remote sensing for dust susceptibility characterization and biological soil crusts for dust susceptibility reduction. Doctoral dissertation, Michigan Technological University

